# Omega-3 from Flaxseed Oil Protects Obese Mice Against Diabetic Retinopathy Through GPR120 Receptor

**DOI:** 10.1038/s41598-018-32553-5

**Published:** 2018-09-25

**Authors:** Marcella Neves Dátilo, Marcella Ramos Sant’Ana, Guilherme Pedron Formigari, Patrícia Brito Rodrigues, Leandro Pereira de Moura, Adelino Sanchez Ramos da Silva, Eduardo Rochete Ropelle, José Rodrigo Pauli, Dennys Esper Cintra

**Affiliations:** 1 Nutritional Genomics Laboratory, LabGeN, School of Applied Sciences, UNICAMP, São Paulo, Brazil; 2 Laboratory of Molecular Biology of Exercise, LabMEx, School of Applied Sciences, UNICAMP, São Paulo, Brazil; 3 School of Physical Education and Sport of Ribeirão Preto, USP, São Paulo, Brazil; 4 Nutrigenomics and Lipids Research Center, CELN, School of Applied Sciences, UNICAMP, São Paulo, Brazil

## Abstract

The chronic and low-grade inflammation induced by obesity seem to be the “*first hit*” to retinopathy associated to diabetes type 2. Herein, we hypothesized that omega-3 fatty acids from flaxseed oil enriched diet disrupt the pro-inflammatory status in the retina, protecting against retinopathy development. For eight weeks under a high-fat diet (HF), several physiological parameters were monitored to follow the metabolic homeostasis disruption. After this period, mice were treated with a HF substituted in part of lard by flaxseed oil (FS) for another eight weeks. Food behavior, weight gain, glucose and insulin sensitivity, electroretinography, RT-qPCR and western blots were carried out. The HF was able to induce a pro-inflammatory background in the retina, changing IL1β and TNFα. VEGF, a master piece of retinopathy, had early onset increased also induced by HF. The FS-diet was able to decrease inflammation and retinopathy and improved retinal electro stimuli compared to HF group. GPR120 and GPR40 (G Protein-Coupled Receptors 120 and 40), an omega-3 fatty acid receptors, were detected in the retina for the first time. FS-diet modulated the gene expression and protein content of these receptors. Thus, unsaturated fatty acids protect the retina from diabetes type 2 mice model from disease progression.

## Introduction

There is a global diabetes epidemic^1^ linked to obesity increase^2^. Once the fat accumulation-induced pro-inflammatory status induces a systemic glucose homeostasis dysfunction, the glucose uptake by skeletal muscle is decreased while gluconeogenesis by the liver is increased^3,4^. Retinal tissue is one of the leading susceptible tissue to acute hyperglycemia because the glucose transporter 1 (GLUT1), the primary glucose transporter in this tissue, is insulin independent and control by glucose active transport^5^.

In the retina, the increased expression of pro-inflammatory proteins and growth factors could be induced by high glucose levels^6^, saturated fatty acids from diet^7^, or lipolysis of adipose tissue (free fatty acids)^8,9^ through Toll-Like Receptor (TLR) and interleukin 1 receptor (IL1R) pathways. VEGF (Vascular and Endothelial Growth Factor) is considered the main marker involved in retinal damage, once the enhance of intraocular inflammatory process^10,11^ by the increase of vessel-permeability^12,13^ results in pathological angiogenesis, which is irregularly distributed with poorly constructed vessels prone to leak, leading to fluid build-up within the retina^14^. In the long term, these changes are capable of leading to apoptosis of retinal neurons^15^. The VEGF inhibitors such as aflibercept and ranibizumab have demonstrated enormous benefits to patients with diabetic retinopathy^16^. However, these drugs show a high financial cost^17^ and refractoriness^18^. Therefore, different strategies need to be explored to control or postpone the disease progression.

Omega-3 (ω3) fatty acids, which is obtained from marine sources [Eicosapentaenoic acid EPA (C20:5) and Docosahexaenoic acid DHA (C22:6)]^19^ or vegetable oils [Alpha-linolenic acid ALA (C18:3)]^20^ have their anti-inflammatory properties extensively investigated on the whole body^21^, including in the retina^22,23^. In contrast, the deficient intake of ω3 contributes to retinal degeneration, once ω3 is an essential fatty acid in mammals and retinal composition^24^. Thus, some clinical trials evaluated this hypothesis. The AREDS2 study enrolled 4203 elderly participants at high risk of progression to advanced age-related macular degeneration, which received 1 g of ω3 for five years. In the primary analysis, there was no reduction of disease progression^25^. On the other hand, the PREDIMED study enrolled 3482 participants receiving at least 500 mg of dietary ω3 for six years. In this approach, the authors found a decreased risk of sight-threatening in diabetic retinopathy^26^.

In the present study, we evaluated the recently described G-Protein Coupled Receptor 120 (GPR120), the main receptor of ω3, which mediates its potent anti-inflammatory and insulin-sensitizing receptor responses^21,27,28^. The ω3-sensitized GPR120 activate and recruit the first downstream mediator (β-arrestin2 protein), which concomitantly recruits TAB1/2 from TLR2/4 and TNF-α pathways, disassembling these inflammatory cascades^23^ by TAK1 dephosphorylation, a nodal protein. Our research group has investigated this mechanism in several tissues such as liver, skeletal muscle, adipose^27^, aorta^29^, and hypothalamus^28^; however, under this point of view, the retina remains completely unexplored.

The comprehension of molecular mechanisms modulated by ω3 fatty acids in the retinal tissue under hyperglycemic and pro-inflammatory state could contribute to increasing the knowledge regarding non-pharmacological treatment approaches.

## Results

### GPR120 activation by time course test

**Figure 1:**
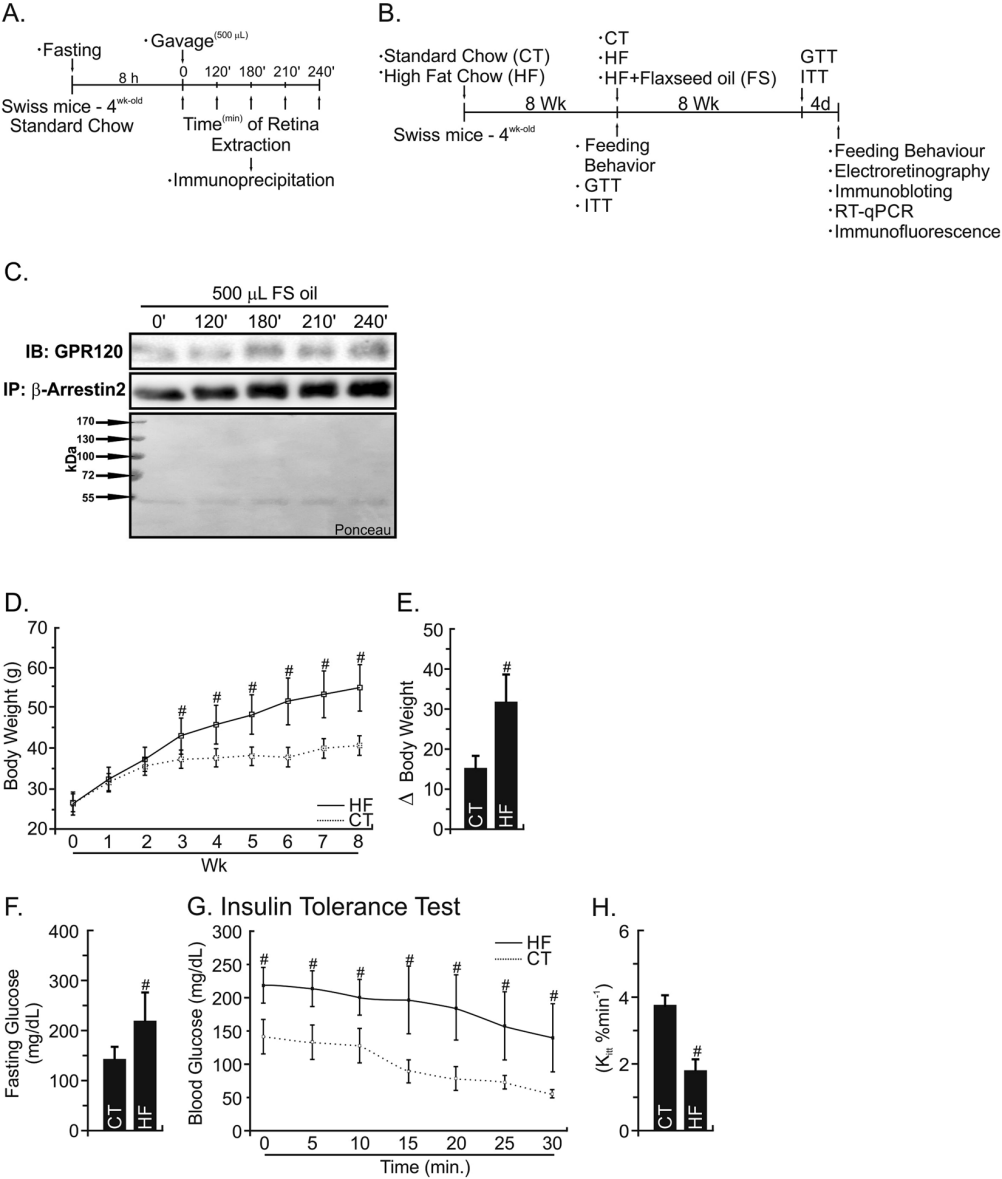
Experimental Protocol Design and Metabolic Characterization. (**A**) 4-week old male Swiss mice fasted for 8 h. Mice were separated into 5 groups and gavaged with 500 µL of flaxseed oil. The retinal tissue was removed immediately after gavage (0 minutes) or after 120; 180; 210 and 240 min. (**B**) 4-week old male Swiss mice were fed with regular chow (CT) or high-fat diet (HF) for 8 wk. The insulin and glucose sensitivity tests (ITT/GTT) were carried out. After to ensure the obesity and diabetes condition, mice were separated into three groups and maintained in CT diet or HF diet in accordance with the groups: CT (CT); HF (HF); HF substituted in 10% of lard by flaxseed oil (FS). At the end of experimental period, GTT and ITT test, feeding behavior, electroretinography, immunoblotting, immunoprecipitation RT-qPCR and immunofluorescence were carried out. (**C**) To test GPR120 intracellular signaling cascade, extracts obtained from the retina were used in immunoblotting (IB) experiments. Samples containing 0.3 mg total protein extract were incubated with β-arrestin2 and the immunocomplexes were recovered with protein A-Sepharose and separated by SDS-PAGE, transferred and blotted (IB) with GPR120 antibody. Loading was evaluated by re-probing the membranes with anti- β-arrestin2. Ponceau staining was adopted to improve and guarantee the quality of western blot membrane running. N = 3/group. (**D**) Teeding behavior and body mass were determined throughout the experimental period. (**E**) The delta between the beginning (1^st^ wk) and last-wk (8^th^ wk) of body weight evaluation. (**F**) Fasting glucose and (**G**,**H**) the glucose decay during an ITT test (%/min) was obtained at the end of an eight-week experimental period for Swiss mice fed regular chow-diet (CT) or high-fat diet (HF). N = 5. *P < 0.05 vs. CT by Bonferroni’s test.

To test the ability of ω3 fatty acids, presented in its natural food matrix (flaxseed oil), to cross the blood-retinal barrier and activate its receptor (GPR120), a time course test was performed. Lean Swiss mice were gavaged with 500 μL of flaxseed oil and the retina was removed at different times for immunoprecipitation. We observed ω3 was able to activate GPR120 receptor through the interaction with β-arrestin2 protein after 180 minutes post-gavage (Fig. 1C). This approach allowed us to proceed with the next experiments with flaxseed oil added in the experimental diets.

### High-fat diet induces body weight gain and insulin resistance

Four weeks under HF-diet was enough to increase the body weight in comparison with the CT group (P < 0.05) (Fig. 1D). At the end of 8 weeks, this difference was intensified (Fig. 1D and E) and the HF-diet was able in worsening fasting serum glucose (Fig. 1F) and insulin tolerance (Fig. 1G) by decrease the constant of glucose decay (Fig. 1H) when compared to the CT group (P < 0.05).

### FS oil Substitution Changes the Blood Fatty Acid Profile

To ensure that ω3-alpha-linolenic fatty acid (C18:3) achieved efficiently the bloodstream after treatments with FS diet, we carried out the blood fatty acid profile (Table 1). C18:3-ω3 was 130% higher in FS group in comparison to HF (P < 0.05). Another ω3 species, such as C20:5 (eicosapentanoic-EPA) and C22:6 (docosahexanoic-DHA) accompanied this increment in 160% and 158% respectively when compared to HF group (P < 0.05). The sum of total ω3 fatty acids was 44% higher than HF group (P > 0.05). As expected, the total saturated fatty acids decreased 9.4% in the FS group when compared to HF (P < 0.05). The arachidonic fatty acid (ω6 C20:4) was increased in the HF group when compared to CT or FS groups. The sum of total ω6 fatty acids in the FS group was lower (9.1%) when compared to HF (P < 0.05).

**Table 1:** Fatty Acids Profile from Blood.

Fatty Acids	CT	HF	HF + FS
C12:0	0.06 ± 0.02	0.03 ± 0.01^#^	0.04 ± 0.02
C14:0	0.24 ± 0.05	0.14 ± 0.01^#^	0.16 ± 0.02
C16:0	20.87 ± 0.86	21.11 ± 0.52	19.01 ± 0.72*
C18:0	8.91 ± 0.67	12.52 ± 0.96^#^	12.62 ± 1.48
Ʃ SFA	30.09	33.80^#^	31.84*
C16:1 ω7	1.95 ± 0.23	0.70 ± 0.05^#^	0.72 ± 0.12
C18:1 ω9	17.13 ± 0.89	16.44 ± 0.92	16.82 ± 1.79
C20:1	0.45 ± 0.12	0.22 ± 0.20^#^	0.42 ± 0.03*
Ʃ MUFA	19.54	17.36^#^	17.97
C18:2 ω6	27.76 ± 0.49	19.66 ± 1.76^#^	24.86 ± 1.70*
C18:3 ω6	0.19 ± 0.03	0.16 ± 0.02	0.10 ± 0.03*
C20:2 ω6	0.34 ± 0.14	0.16 ± 0.11^#^	0.25 ± 0.02*
C20:3 ω6	1.54 ± 0.10	2.20 ± 0.28^#^	2.26 ± 0.50
C20:4 ω6	15.77 ± 1.11	22.61 ± 0.43^#^	13.69 ± 2.41*
Ʃ ω6	45.61	44.79	41.16*
C18:3 ω3	0.52 ± 0.08	0.10 ± 0.02^#^	1.33 ± 0.21*
C20:5 ω3	0.15 ± 0.03	0.10 ± 0.01^#^	1.60 ± 0.09*
C22:6 ω3	4.10 ± 0.29	3.85 ± 0.67	6.11 ± 0.58*
Ʃ ω3	4.77	4.05	9.03*
Ʃ PUFA	50.38	48.85^#^	50.19
ω6:ω3 ratio	9.56	11.06	4.56

CT-Control group; HF-High-fat group; HF + FS-Flaxseed group. SFA-Saturated Fatty Acids; MUFA-Monounsaturated Fatty Acids; PUFA-Polyunsaturated Fatty Acids. Data are present as mean ± standard deviation. N = 5 per group. ^#^vs. CT. *vs. HF group by Bonferroni’s test (P < 0.05).

### FS oil substitution improves the glucose homeostasis without body weight changes

**Figure 2:**
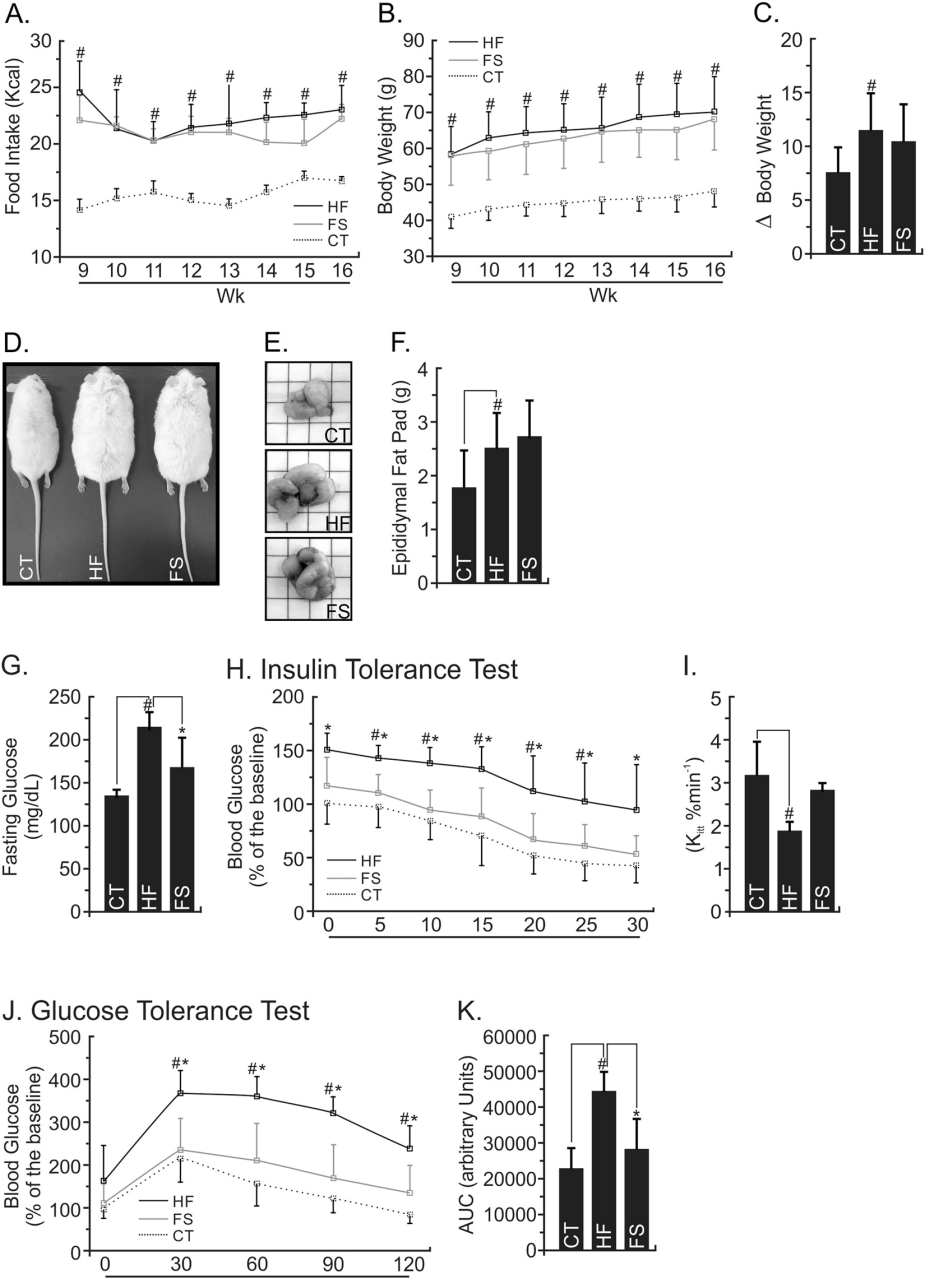
Effects of FS diet on the Metabolic Parameters of Obese and Diabetic Mice. (**A**) Food intake and (**B**) body mass gain for each group during the 8 last weeks under experimental diets. (**C**) The delta between the beginning (9^th^ wk) and last-wk (16^th^ wk) of body weight evaluation (**D,E**) mice illustrative and representative photographs at the end of experimental period and (**F**) average of the right flank of epididymal mice fat pad. At the end of the experimental period, the mice were submitted to (**G**) fasting glucose analysis, (**H**) insulin tolerance test (ITT) and (**I**) constant of glucose decay; (**J**) glucose tolerance test (GTT) and its (**K**) area under the curve. In all experiments, n = 5; ^#^P < 0.05 vs. CT. *P < 0.05 vs. HF (Bonferroni’s test).

The total of energy (Kcal) intake was increased on HF group when compared to the CT (Fig. 2A), however, FS diet did not change it when compared to the HF (P > 0.05). The HF group had an increment in body weight (Fig. 2B), accumulated body weight (Fig. 2C) representative photographs of mice body size (Fig. 2D) and epididymal fat pad (Fig. 2E); and the weight of epididymal adipose tissue (Fig. 2F) when compared to the CT (P < 0.05). However, the replacement of 10% of lard by flaxseed oil in the diet of FS group was not enough to change body weight or composition compared to the HF group (P > 0.05) (Fig. 2B–D). The HF-diet even worsen the glycemic homeostasis leading to a significant increase in fasting blood glucose (Fig. 2G), insulin resistance (Fig. 2H and I) and glucose intolerance (Fig. 2J and K) when compared to the CT group (P < 0.05). Interestingly, FS-diet improved fasting blood glucose (Fig. 2G) and the GTT area under the curve (Fig. 2J and K) when compared to the HF group (P < 0.05).

### Evaluation of retinal function by full-flash ERG

**Figure 3:**
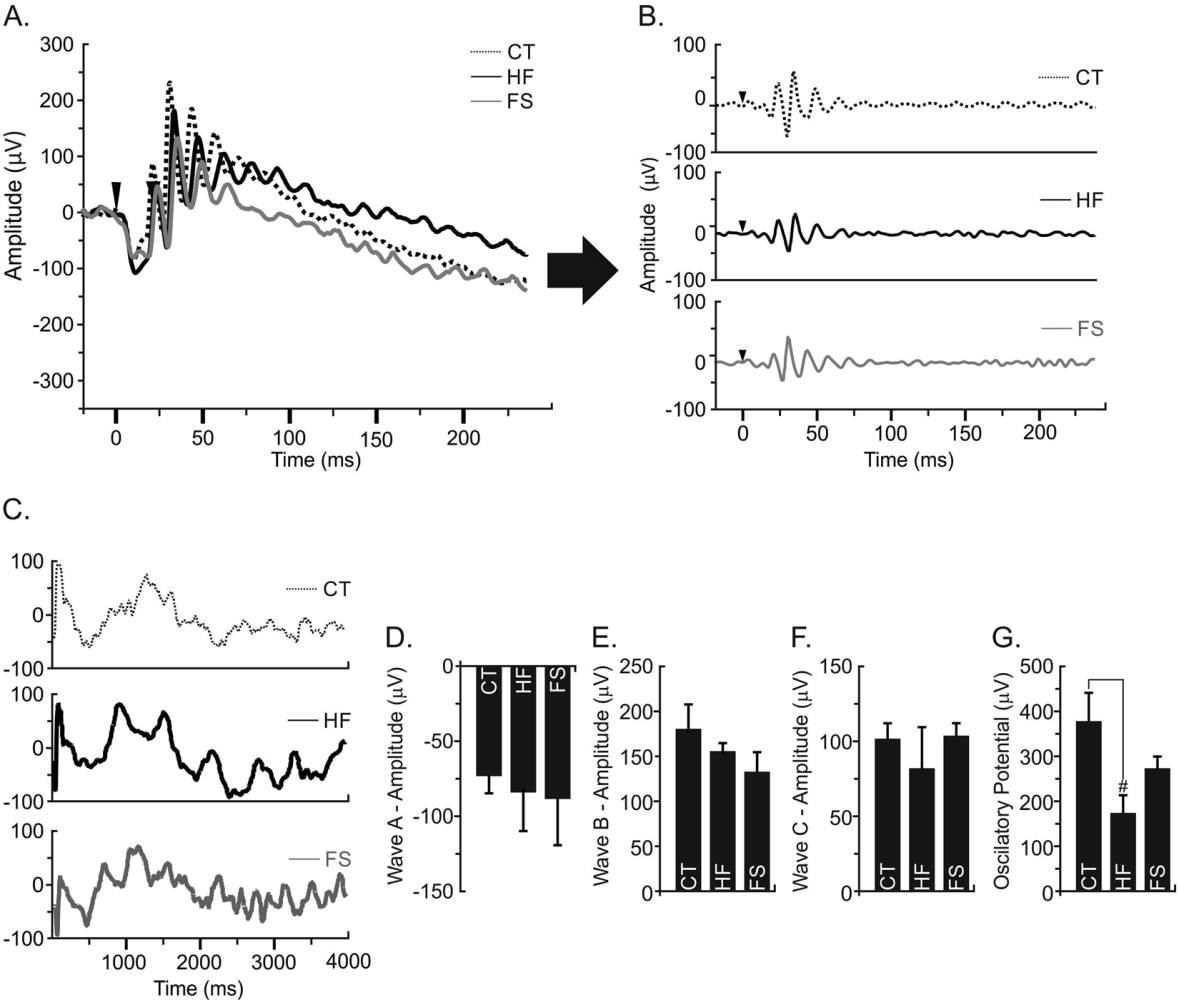
Retinal Electroretinography Responses (RER). (**A**) Representative image of waves A and B from RER on different experimental groups. (**B**) Representative image of the oscillatory potential at 0.25 cd.s/m^2^ in experimental groups. (**C**) Representative image of waves C. (**D**) Wave A amplitude. (**E**) Wave B amplitude. (**F**) Wave C amplitude. (**G**) Oscillatory potential. In all experiments, n = 6; ^#^P < 0.05 vs. CT.

To determine whether the exposure for 16 weeks to experimental diets was able to change the retinal function, an electroretinogram (ERG) was performed. After exposition to different luminous intensities, even under high body weight and glucose levels, HF group did not show differences among amplitudes of wave A, B or C (Fig. 3A–C and E), which are responsiveness for cones, rods, and epithelial cells, at the end of experimental period, when compared to the CT group (P > 0.05). However, mice under HF decreased the oscillatory potential (Fig. 3G) suggesting neural retina damages, when compared to the CT group (P < 0.05). On the other hand, FS group was protected from retinal damage induced by HF-diet, suggesting benefits linked to the ω3 fatty acids (Fig. 3G).

### GPR120 and GPR40 are expressed and extensively distributed in the retinal tissue

**Figure 4:**
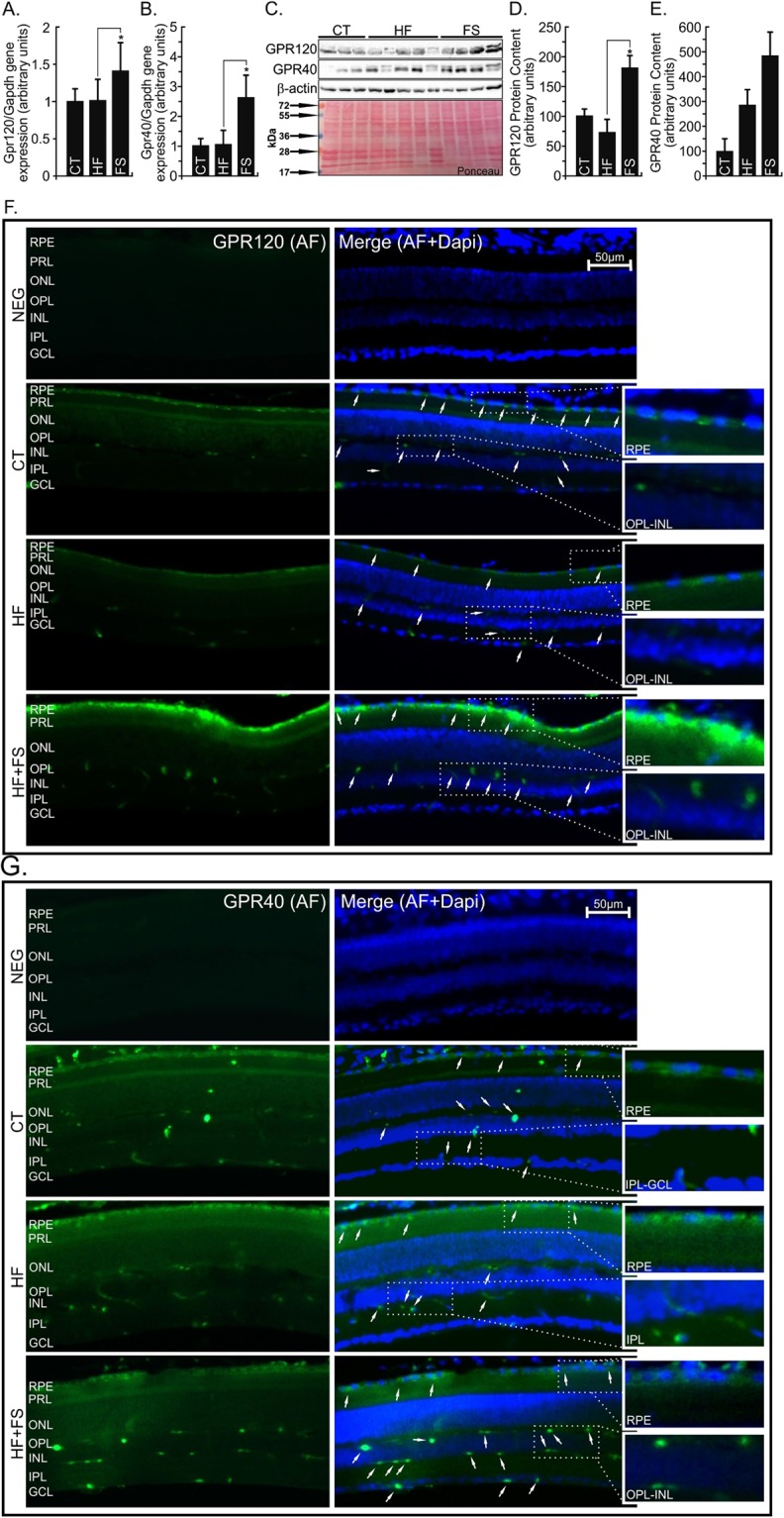
GPR120 and GPR40 Gene Expression, Protein Content and Immunohistochemistry Mapping in the Retina. (**A,B**) Total RNA obtained from retina was used in real-time q-PCR to amplify the mRNAs of GPR120 **(A)** and GPR40 (**B**). Gapdh was adopted as an internal control. n = 8. (**C–E**) Protein extracts obtained from the retina of Swiss mice were used in immunoblotting (IB) experiments, to evaluate GPR120 and GPR40 protein contents. Specific antibodies against GPR120 or GPR40 were used. Loading was evaluated by re-probing the membranes with anti- β-actin. The images from WB gels are original and did not cropped. Ponceau staining was adopted to improve and guarantee the quality of western blot membrane running. N = 5 per group. ^#^vs. CT. or *vs. HF groups by Bonferroni’s test (P < 0.05). (**F,G**) Retina from Swiss mice fed regular chow (CT), high-fat (HF) or flaxseed oil- (FS) substituted (10%) diet were used in immunofluorescence experiments. Specific antibody against GPR120 or GPR40 (Green AlexaFluor546) was performed in 20-µm sections of the retina used to identify the respective protein localization targets. The photomicrographs were taken on 50x magnification, and on the 100x highlighted image. AL-Alexa Fluor 546; NEG-Negative control (GPR120 or GPR40 peptide blocking); RPE-Retinal Pigment Epithelium; PRL-Photoreceptor Layer; ONL-Outer Nuclear Layer; OPL-Outer Plexiform Layer; INL-Inner Nuclear Layer; IPL-Inner Plexiform Layer; GCL-Ganglion Cell Layer. White arrows indicate GPR120 or GPR40 position.

To evaluate the presence and distribution in the retinal tissue of ω3 fatty acids receptors, we carried out three different approaches such as mRNA quantification by RT-qPCR, protein content by Western blot, and immunohistochemical photomicrography. The GPR120 and GPR40 were found in the retina of mice under all treatments (Fig. 4A and B); however, FS diet modulated the GPR120 and 40 gene expression in comparison to HF group (P < 0.05). The GPR120 protein was detected in the retinal of mice under both CT or HF and FS treatments. However, the FS-diet positively modulated this receptor in comparison with the HF group (P < 0.05) (Fig. 4C and D). The GPR40 protein was detected by immunoblots, but without statistic difference (P > 0.05) among groups (Fig. 4E). Interestingly, immunofluorescence showed us an extensively GPR120 (Fig. 4F) or GPR40 (Fig. 4G) receptors distribution over different retinal layers. However, GPR120 was concentrated in the retinal pigment epithelium (RPE), outer nuclear (ONL) and inner nuclear layers (INL), while GPR40 was concentrate also in the outer plexiform (OPL) and ganglion cell layers (GCL).

### FS reduces retinal inflammation in obese and type 2 diabetes mice model

**Figure 5:**
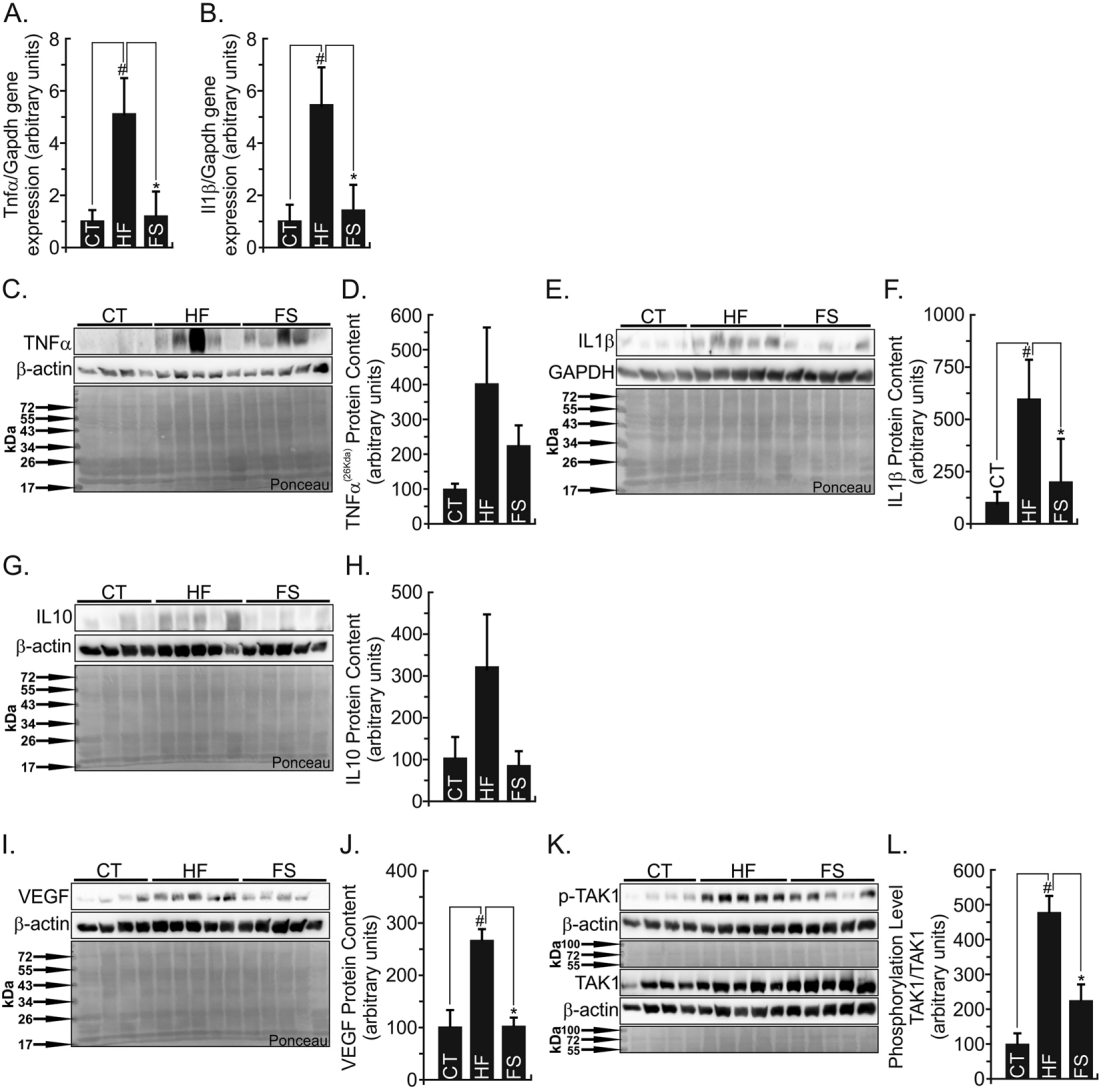
Inflammatory and Retinopathy Gene and Protein Markers. (**A,B**) Total RNA obtained from retina was used in real-time q-PCR to amplify the mRNAs of TNFα **(A)** and IL1β (**B**). Gapdh was adopted as an internal control. n = 5. Protein extracts obtained from the retina of Swiss mice were used in immunoblotting (IB) experiments, to evaluate pro- and anti-inflammatory protein contents. Specific antibodies against TNFα (**C,D**), IL1β (**E,F**), IL10 (**G,H**), VEGF (**I,J**), and phosphor- TAK1 (**K,L**). Loading was evaluated by re-probing the membranes with anti- β-actin or GAPDH or TAK1. The images from WB gels are original and did not cropped. Ponceau staining was adopted to improve and guarantee the quality of western blot membrane running. N = 5 per group. ^#^vs. CT. *vs. HF group by Bonferroni’s test (P < 0.05).

After 16 weeks of CT, HF- or FS-diets consumption, the pro- and anti-inflammatory markers were evaluated in the retina. The HF-diet induced inflammatory status, increasing significantly TNFα and IL1β gene expressions (P < 0.05) when compared to the CT group (Fig. 5A and B). This behavior was similar (P < 0.05) for the protein levels (Fig. 5C–F). The partial replacement of saturated fat source (HF) for unsaturated fatty acids (FS) was able to protect retina against the increase of TNFα and IL1β gene expressions augment (P < 0.05) (Fig. 5A and B). Also, both TNFα and IL1β protein levels were decreased (P < 0.05) (Fig. 5C–F). Interleukin 10 was not changed by the different diets (P > 0.05) (Fig. 5G and H).

Interestingly, 16 weeks under HF-diet were enough to increase the primary retinopathy marker, VEGF (P < 0.05) when compared to the CT group (Fig. 5I and J). Alson, FS-diet protected retinal tissue from the VEGF increment (P < 0.05) when compared to the HF group (Fig. 5I and J). Finally, HF-diet was able to phosphorylate and activate the Tak1 protein (P < 0.05), which is crucial for NF-kB cascade activation (Fig. 5K and L). The FS-diet reduced Tak1 phosphorylation (P < 0.05), showing the anti-inflammatory effect of ω3 fatty acids occurs through GPR120 (Fig. 5K and L).

## Discussion

The chronic and low-grade inflammation processes contribute to the development of the early stages of diabetic retinopathy. Several studies have implicated ω3 fatty acids in the improvement of physiological and molecular characteristics in the retinal tissue. However, these investigations did not define the action mechanisms of the ω3 fatty acids^19,22–24^. We tested an oral treatment protocols, with a rich and natural ω3 source (flaxseed oil) from diet, mimicking a real and rational possibility for being used by human beings, once fish oils today, unfortunately, could be contaminated by heavy metals (e.g. mercury)^30^.

Then, the primary aim of the present study was to determine the presence and functionality of ω3 receptors, GPR120 and GPR40, in the retina on the basal state. The GPR120 and GPR40 mRNAs (Fig. 4A and B) and protein contents (Fig. 4C–G) were detected in the retinal tissue of lean mice. However, only GPR120 (Fig. 1C), but not GPR40 (data not showed), was responsive to ω3 stimuli by the β-arrestin2 binding test, in a time-dependent manner (180 min). This observation is in agreement with previous reports for tissues such as skeletal muscle, adipose tissue, liver^21^, aorta^29^, hypothalamus^28^, and renal cells^27^. Again, this is the first approach adopted to the retina. Connor *et al*.^22^ and Tikhonenko *et al*.^23^ treated mice with ω3 enriched diet and observed the increase in ω3 fatty acid profile content in the retina, but without functional tests for GPR120/40 receptors.

Before to test the ω3 anti-inflammatory hypothesis in the retinal tissue, we induced mice to the obesity and hyperglycemic state through an HF-diet (Fig. 1D and E). Swiss mice are a prone strain^31^, able to mimic obesity comorbidities highly similar to humans^32^. Herein, compared to the CT-group, mice under HF-diet exposure during 8 weeks increased body mass and fasting blood glucose with consequently insulin resistance (Fig. 1F–H). After intervention period, the alpha-linolenic fatty acid in the diet containing 10% of flaxseed oil was bioavailable in the bloodstream. The ω6:ω3 ratio in the FS diet was suitable when compared to other groups, once elevated ratios for ω6 instead of ω3 is an independent proinflammatory factor^33^. We observed an increment on ω6 (arachidonic fatty acid) bioconversion in the HF group when compared to FS or CT groups (P < 0.05). It could, adjacently to saturated fatty acids on HF-diet, to increase the pro-inflammatory status observed in the retina. Interestingly, the alpha linolenic fatty acids from FS group was partially bioconverted in other ω3 fatty acids longer ones (EPA and DHA), decreasing inflammation, such as suggested by Tikhonenko^23^ and Johansson^34^ groups; and reinforced by this work.

Then, the FS diet was able to reduce fasting blood glucose and glucose intolerance, improving whole body glucose homeostasis (Fig. 2G–K) even without changes in body mass or epididymal fat (Fig. 2B–F). Possibly, a long-term of FS-diet consumption is necessary to change body composition parameters. Several studies showed the role of flaxseed oil and fish oil as therapeutic agents in the improvement of glycemic homeostasis improvement in mice^20,21,27^ and humans beings^35,36^ with obesity and its associated comorbidities. However, Baranowski *et al*. treated mice with dietary flaxseed oil and showed a reduction in the adipocyte size, alterations in several pro-inflammatory molecular parameters even without significant changes in the body weight^20^. These data are exciting once body weight loss could influence several biological parameters. This study (Baranowski *et al*.) reinforces the current findings regarding the positive effects of “ω3 molecule” in inducing, *per se*, biological alterations independently of body weight loss. Again, this statement is emphasized once the FS- and HF-diets were caloric equivalently in this study.

Hyperglycemia is a determinant factor capable of inducing alterations in the retina structure and, consequently, in the visual acuity^6,10,11,37^. In fact, a reduction in the oscillatory potential was evidenced in mice treated with HF-diet, but not in FS, suggesting the compromised visual acuity (Fig. 3G). Here, this model was able to offer dual main characteristics to retinal damage, hyperglycemia, and retinal inflammation. Rajamani *et al*., (2014) explored the increased expression and activity of TLR2 and TLR4 in type 2 diabetic subjects^6^. Additionally, Anan *et al*., (2010) and Tang & Kern (2011) found an association between retinopathy and visceral fat accumulation, attributing the retinopathy to pro-inflammatory state also in humans^8,37^.

The HF-diet induced a pronounced pro-inflammatory state in the retinal tissue, changing the gene expression and protein content of the main pro-inflammatory markers (TNFα and IL1β), and activating the IL10, a homeostatic anti-inflammatory protein (Fig. 5A–H). On the other hand, the FS-diet decreased both TNFα and IL1β gene expressions and protein contents. However, as a highlight of this study, we emphasize the VEGF modulation by both diets, once this growth factor is a determinant molecule involved in the diabetic retinopathy^10–14^. In general, scientific literature reports VEGF as a late disease marker, which is firstly dependent on biochemical and hemodynamic alterations. The retinal endothelial cells have a high significantly amount of VEGF receptors, and the hypoperfusion and hypoxia induce VEGF expression. Therefore, VEGF is considered as a master angiogenesis regulator and a pro-inflammatory protein, leading to permeability by rapid tight-junctions’ (occludin and zonula occluden1) phosphorylation and disable^13^. The FS-diet was able to protect retina against VEGF raises.

Also, VEGF gene is also controlled by NF-κB^10,38^. However, NF-κB is strongly regulated by a nodal TAK1 protein cascade, which is upstream activated by juxtamembrane-adjacent TLR, TNFα, and interleukin (IL1β and IL6) receptor proteins^27,39^. Herein, the ω3 in the flaxseed diet activated GPR120, which recruited β-arrestin2 and reduced TAK1 phosphorylation, disassembling the pro-inflammatory cascade triggered by high saturated fatty acids in the HF-diet.

For the first time, both GPR120 and 40 were, through different approaches, identified, quantified and mapped in the retinal tissue (Figs 1C, 5A–E and 4F and G, respectively). Retinal pigment epithelium (RPE), outer nuclear (ONL), and inner nuclear layers (INL) concentrate GPR120 receptor (Fig. 4F), while GPR40 (Fig. 4G) was also found in the outer plexiform layer (OPL). In a summarized retinal layers overview; a) RPE cells are involved in the regeneration and repair of photoreceptor cells^40^; b) The normal ONL contains rod and cone photoreceptor nuclei throughout the retina^41^; c) INL consist of bipolar cells, which are directly synapsed to retinal ganglion cells and Müller glial cells^42^; d) and OPL has a certain degree of integration of the visual message takes place at the first synapse in the retina^43^.

Interestingly, in the basal state, RPE cells are involved in the production of VEGF^44^, monocyte chemotactic protein (MCP)-1, IL8, IL6, and interferon (IFN)-γ with or without stimulation by other pro-inflammatory cytokines^45^. However, overexpression of cytokines can impair its special features, causing retinal degeneration and even blindness^40^. Once controlling the pro-inflammatory cytokines background, GPR120 expressed in this layer could contribute to RPE function, maintaining ONL and INL layers integrated.

OPL has integrative neurons and forms synapses to the bipolar, amacrine, and Müller glial cells in INL, which is closely integrated to ONL^43^. The ONL and INL thickness provide an indirect measure of photoreceptor survival^41^; however, INL directly synapses to retinal ganglion cells and Müller glial cells, which are involved in retinal environment homeostasis. Retinal tissue is structured similarly to the central nervous system (CNS) and, for example, in the hypothalamus, GPR120 is expressed and recognizes ω3 fatty acids, disassembling inflammatory tonus induced by obesity state or triggered directly by saturated fatty acids^28^. As well in the CNS, Müller glial cells are throughout the main retinal layers and could also be responsive to fatty acids from the diet, controlling the local immune response and, consecutively, structural tissue integrity. The GPR120 receptor was also described in the liver, in the surface of Kuppfer cells, and in macrophages of the aorta endothelium, decreasing pro-inflammatory cytokines, reverting hepatic steatosis^21^ and atherosclerosis^29^, respectively, when mice were treated with flaxseed oil.

Despite the presence of GPR40 be present in retinal tissue (Fig. 4B,C,E and G), this receptor did not show significant effects in this study. However, it essential to clarify that the homology between GPR40 and GPR120 is only 10%^46^. This percentage allows to GPR40 to be responsiveness for ω3 fatty acids^21^; however, without biological impact for this model. GPR40 is highly responsiveness to oleic fatty acid (ω9), and its role investigation remains an interesting issue.

**Figure 6:**
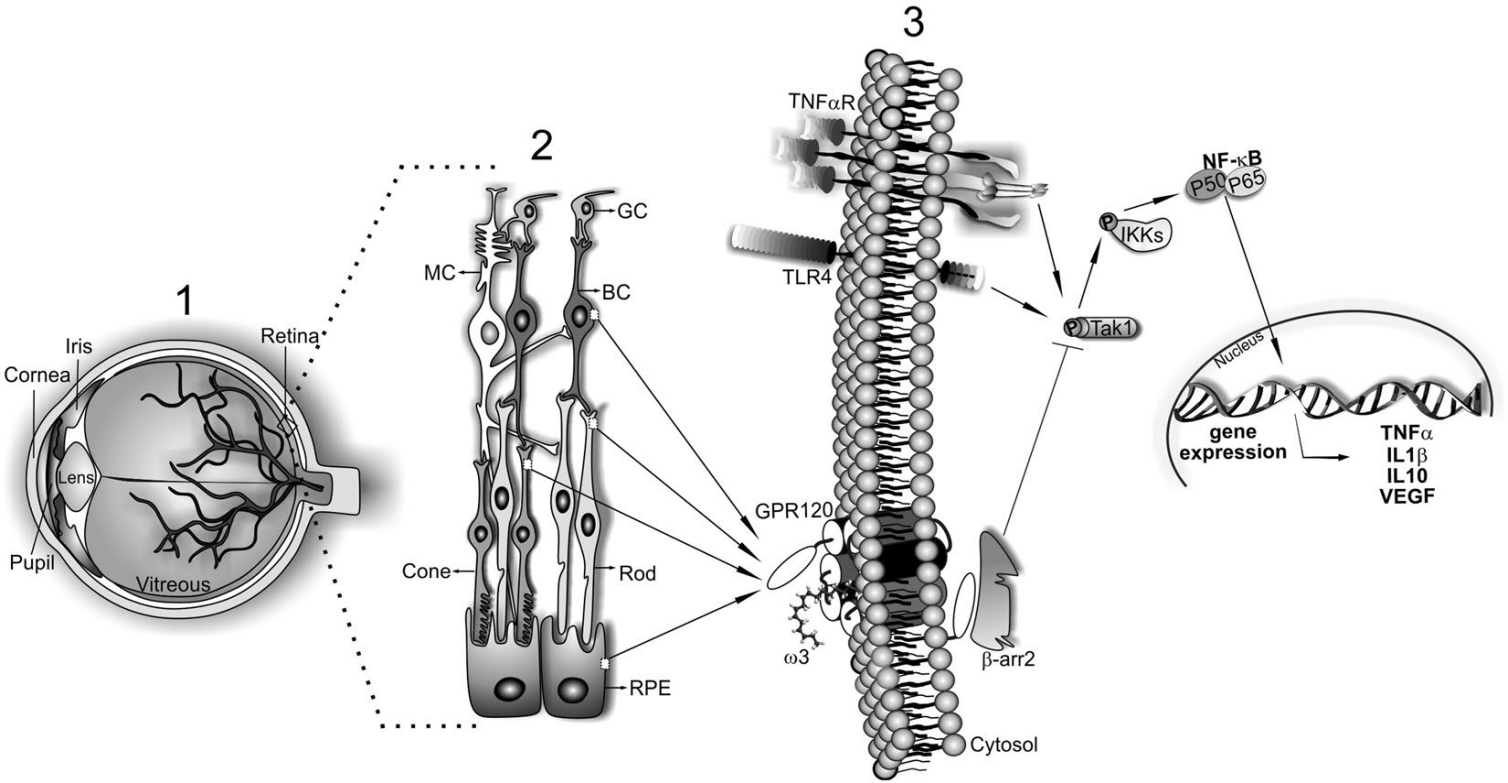
Graphical Abstract. (1) The general eye structure. (2) The main cellular types involved in the retinal structure: MC microglial cells; GC ganglion cells; BC bipolar cells; RPE retinal pigmented cells; Cones and Rods. (3) The pro-inflammatory signaling triggered by both TLR4 or cytokine receptors such as TNFα activates Tak1. These proteins recruit three Tab monomers, which phosphorylates and activates Tak1. Tak1 is a nodal molecule responsible to activate the inhibitor of kinase kappa (IKκ) and consequently the canonical pro-inflammatory pathway orchestrated by NF-κB. On the other hand, omega-3 fatty acid from flaxseed oil activates GPR120 receptor in the cell membrane. GPR120 recruit beta-arrestin2 protein which detached the protein complex formed by Tab and Tak1 inhibiting the pro-inflammatory cascade.

Altogether, these results describes an essential part of the role of GPR120, a central ω3 fatty acids receptor, such as summarized in the schematic Fig. 6. Although some studies previously described the potential actions of ω3 in the retina of animals and humans, this is the first investigation displaying a mechanistic perspective. Using a nutrigenomics approach, we describe the effects of ω3 molecular actions in decreasing the retina’s inflammatory tonus. Probably, diets containing ω3 fatty acids will never reestablish the visual damage; however, the modulation of diet or the use of chemical GPR120 agonists can deaccelerate the disease progression. As there is a conundrum related to fundamental questions regarding retinal layers and nutrients action, this research field is an opened avenue for future explorations.

## Methods

### Animals

Swiss male mice (4 week-old) were provided by the University of Campinas Animal Breeding Center (CEMIB). All animal procedures were performed in accordance with guidelines and regulations, and approved by University of Campinas Ethical Committee (protocol #4197-1). The animals were housed in individual cages under 12 h dark/12h-light cycle, room temperature at 21 ± 2 °C, and water and diet *ad libitum*. The number of mice used in each experiment is detailed in subtitles of graphs.

### Experimental design

#### Acute GPR120 Activation Test

Firstly, we tested whether there are GPR120 in the retina tissue and its activation time. Then, ω3 fatty acids from flaxseed oil was administered to mice (4 week-old), after 8 hours of fasting. The retina was removed immediately after administration (group 0 min) or after 120, 180, 210 or 240 min. GPR120 from retina was submitted to immunoprecipitation (please, see immunoprecipitation method) with β-arrestin2 (Fig. 1A).

#### Obesity Induction Protocol

Firstly, mice were randomly distributed into two groups: CT-group, fed a chow diet (standard rodent diet Nuvilab^®^) or HF group, fed with a high-fat diet (35% of total fat), for eight weeks. The high-fat diet was prepared in according to the American Institute of Nutrition (AIN-93G) guidelines^47^, modified to contain 35% of fat^28^ (4% soy oil and 31% of lard Table 2). The body weight was measured once per week and food intake every three days. At the end of this period, the body weight gain, insulin resistance and glucose intolerance were determined. Then, the obese and insulin resistant mice were redistributed in two new groups, in according to Z-score test: a high-fat diet (HF) and flaxseed diet (FS). In this last group, 10% of fat fraction from lard was substituted by 10% of flaxseed oil^21,28^. The pure flaxseed oil was previously analyzed by mass spectrometry chromatography, and the alpha-linolenic (C18:3) fatty acid content reached 52.3%. Therefore, the diets were prepared every seven days and maintained safely of light exposure at 4 °C. These groups were maintained in this protocol for another eight weeks. The control group was maintained in chow diet (CT) (Fig. 1B).

**Table 2:** The Composition of the Experimental Diets.

Ingredients	HF (g/Kg^−1^)	HF + FS (g/Kg^−1^)
Corn Starch	115.5	115.5
Casein	200	200
Dextrinated Starch	132	132
Sucrose	100	100
Cellulose	50	50
Soybean Oil	40	40
Flaxseed Oil	—	104
Lard	312	208
Vitamin Mix	10	10
Mineral Mix	35	35
L-Cysteine	3	3
Choline	2.5	2.5
Total	1000	1000

Diet based on AIN-93 G – American Institute of Nutrition^47^. HF – high-fat.

### Experimental Procedures

#### Intraperitoneal Insulin Tolerant Test (ip.ITT) and Glucose Tolerant Test (ip.GTT)

These tests were carried out after eight hours of fasting and in two moments: 1) at the end of the obesity period induction, and 2) at the end of the experimental period. For ip.ITT, 1,5U/kg body weight^−1^ of insulin (Humulin R^®^ - Lilly Indianapolis USA) was injected intraperitoneally (i.p.). Blood samples were collected at 0, 5, 10, 15, 20, 25 and 30 min from the tail vein for serum glucose determination in the electronic glucometer (Optium Xceed^®^ - Abbott). The constant for the rate of serum glucose disappearance was calculated using the formula 0.693/biological half-life (*t ½*). The plasma glucose *t ½* was calculated from the slope of the last square analysis of plasma glucose concentration during the linear phase of decline, as previously described^48^.

For ip.GTT, after 8 hours of fasting, a glucose solution (2 g/Kg body weight) was administered i.p. Blood samples were collected at 0, 30, 60, 90 and 120 min from the tail vein for serum glucose determination. Results were presented as the area under the curve.

#### Electroretinography

At the end of the experimental period, animals were submitted to full flash electroretinography. Thus, the animals were previously anesthetized with ketamine and xylazine (75 and 7.5 mg/kg^−1^, respectively) under dim red illumination (λmax = 650 nm). Retinal function was measured using the UTAS-E3000 system (LKC Technologies Inc., Gaithersburg, MD, USA) as previously described^49^. The pupils were dilated with tropicamide (Mydriacyl 0.5%; Allergan, Irvine, CA, USA). The measurements were taken after dark overnight adaptation. An intensity-response series was recorded using a set of ganzfeld flashes with 0.25 cd.s/m^2^ intensity. Recordings were amplified and digitized using a 24-bit A/D converter band passed from 0.3 to 300 Hz with a 50-Hz notch filter.

#### Retina extraction

After 8 hours of fasting, the animals were anesthetized with i.p. injection of ketamine (50 mg/Kg) and xylazine (20 mg/Kg). The anesthesia was assured by the loss of corneal reflexes. After this, the eyeballs were removed. With the aid of a magnifying glass (Leica, M690 model) and ophthalmic scalpel, an incision was made between the iris and internal ocular structures such as vitreous and crystalline. After this, the vitreous and crystalline were removed. Subsequently, with the aid of a forceps, the retina was removed, snap-frozen in liquid nitrogen, and stored at −80 °C until further analysis. In the end, mice were euthanized with anesthetics deepening.

#### Immunoblotting analysis

The retinas were homogenized in extraction buffer (1% Triton X-100, 100 mM Tris, pH 7.4, containing 100 mM sodium pyrophosphate, 100 mM sodium fluoride, 10 mM EDTA, 10 mM sodium vanadate, 2 mM phenylmethanesulphonyl fluoride [PMSF and 0.1 mg of aprotinin/ml] at 4 °C with a Sonicator Vibra-cell VCX 130 (Sonics^®^, Connecticut, USA). The lysates were centrifuged at 12.000 rpm and 4 °C (Eppendorf^®^, 5804 R) for 40 minutes to remove the insoluble material. The supernatants were used for protein quantification, using the Bradford method^50^ and 40–60 μg were used for immunoblotting analysis. The samples were incubated at 95 °C for 5 minutes with Laemmili buffer. After this, samples were applied on a polyacrylamide gel for separation by SDS-polyacrylamide gel electrophoresis. Chemicals and buffers were from Bio-Rad (Richmond, USA). The proteins were transferred to nitrocellulose membranes. Binding of the antibody to nonspecific proteins was minimized by pre-incubation of the nitrocellulose membrane in 5% dry milk for 1 hour and then blotted with specific primary antibodies. Specific bands were labeled by chemiluminescence, visualization was performed with a system for chemiluminescence (G:BOX Chemi XRQ Syngene USA) and quantified using the software UN-SCAN-IT gel 6.1. The antibodies anti-βarrestin2 (sc13140), anti-GPR120 (sc48203), anti-GPR40 (sc32905), anti-IL-10 (sc1783), anti-TAK1 (sc7967), anti-VEGF (sc7269), anti-βactin (sc47778), and anti-GAPDH (sc25778) were purchased from Santa Cruz Biotechnologies. Antibody anti-Phospho-TAK1 [Ser412] (#9339) was purchased from Cell Signaling (Danvers MA USA). Antibodies anti-IL-1β (503502) and anti-TNF-α (506102) were purchased from BioLegend (San Diego, USA).

#### Immunoprecipitation analysis

For immunoprecipitation analysis, 350 μg of total protein for retina homogenates were immunoprecipitated with anti-β-arrestin2 using Protein A sepharose beads (GE Healthcare Life Sciences). Precipitates were then analyzed by a Western blot with anti-GPR120 and re-probed with mouse anti-β-arrestin2.

#### RNA extraction and RTq-PCR

Total RNA was extracted from retinal tissue using TRIzol^®^ reagent (LifeTechnologies Carlsbad CA USA). After this, the total RNA was isolated and quantified (Nanodrop 8000, Thermo Scientific, Wilmington, DE, USA) and five microgram of total RNA from each sample were used to perform the digestion of genomic DNA (Recombinant DNase I RNase-free Takara). The cDNA reverse-transcription was performed using 1 µg of total RNA from retinal samples according to the instructions of the kit (High Capacity cDNA Reverse Transcription - Applied Biosystems). The 10 μL reaction mix consisted of 0.25 μL of primers, 3 µL of PCR master mix, 5 μL of template and DEPC water to achieve the final volume. Next, real-time PCR analysis of gene expression was performed in the StepOne Real-Time PCR Systems (Applied Biosystems). FFAR4/GPR120 (Mm00725193_m1), FFAR1/GPR40 (Mm00809442_m1), IL1β (Mm00434228_m1), TNFα (Mm00443258_m1), and GAPDH (Mm99999915_g1) were obtained from ThermoFisher Scientific.

#### Immunohistochemistry Immunofluorescence

The eyeballs were embedded in OCT compound (Sakura Finetek, Torrance, CA, USA) and sections (20 µm) were taken for the analysis. Sections were incubated in blocking solution (1 x PBS with 5% goat serum) followed by 2 hours of incubation at room temperature with primary antibodies against GPR120 (1:50) or GPR40 (1:10). After that, secondary antibodies conjugated to Alexa Fluor (AlexaFluor 546; Thermo Fischer, CA, USA) were applied for 1 hour at room temperature. After PBS washes the sections were mounted with DAPI (#H-1200 Vector Laboratories; Burlingame, CA, USA) for nucleic acid staining in blue. The eyes sections were examined under a fluorescence microscope (Zeiss, Oberkochen, Germany). Digital images were captured using specific software (AxioVision; Carl Zeiss Microscopy, Thornwood, NY, USA).

#### Mass Spectrometry for Lipid Analysis

Lipids from blood were extracted following the proposed by Shirai (2005)^51^. The fatty acids methyl esters were analysed with a gas chromatograph-mass spectrometer (Shimadzu^®^ GCMS-QP2010 Ultra), and a fused-silica capillary Stabilwax column (Restek Corporation, U.S.) with dimensions of 30 m × 0.25 mm internal diameter coated with a 0.25-µm thick layer of polyethylene glycol. The running conditions was followed in accordance with previous study^52^. Sample volumes of 1 µL were injected at 250 °C using a 20:1split ratio. High-grade pure helium (He) was used as the carrier gas with a constant flow rate of 1.0 mL/min. Mass conditions were as follows: ionization voltage, 70 eV; ion source temperature, 200 °C; full scan mode in the 35–500 mass range with 0.2 s/scan velocity.

### Statistical analysis

After normality test (Kolmogorov-Smirnov (KS)) the difference between CT and HF groups was considered higher than 5% (P < 0.05) using Student’s *t*-test. After KS test, to compare CT, HF, and FS groups, one-way ANOVA was carried out and when significant (P < 0.05), Bonferroni’s test was used to compare the groups. All results were presented as the mean ± standard deviation with GraphPad Prism^®^, v5.0. software^28^.

## Electronic supplementary material

Supplementary Information
