# Supplementary material for: Omega-3 from Flaxseed Oil Protects Obese Mice Against Diabetic Retinopathy Through GPR120 Receptor
Source: Sci Rep. 2018 Sep 25;8:14318. doi: 10.1038/s41598-018-32553-5 (PMC6156233; doi:10.1038/s41598-018-32553-5)
Supplement: Supplementary file 1 — Supplementary Information [file 41598_2018_32553_MOESM1_ESM.pdf]

Title: Omega-3 from Flaxseed Oil Protects Obese Mice Against Diabetic Retinopathy Through GPR120 Receptor

Running Title: Omega-3 Protects Mice Against Retinopathy

Marcella Neves Dátilo; Marcella Ramos Sant'Ana; Guilherme Pedron Formigari; Patrícia Brito Rodrigues; Leandro Pereira de Moura; Adelino Sanchez Ramos da Silva; Eduardo Rochete Ropelle; José Rodrigo Pauli; Dennys Esper Cintra.

Supplementary Information

Western Blot Gel Procedures:

All images obtained from WB gels are originals. None WB image was cropped, inverted, grouped or had the luminosity (high-contrast, overexposure) altered. The "Ponceau" membrane staining test was added in each WB graph to increase the gels reliability. Please, note these descriptions in all legends of all WB figures.
